# Comprehensive Geriatric Assessment of Older Patients with Multiple Myeloma: A Prospective Observational Study

**DOI:** 10.3390/cancers17172904

**Published:** 2025-09-04

**Authors:** Paula Sobrini-Morillo, Celia Corral-Tuesta, Carmen Sánchez-Castellano, Tamara Gutiérrez-Blanco, Pablo Palomo-Rumschisky, Claudia Gabriela Álvarez-Pinheiro, María Jesús Blanchard-Rodríguez, José A. Serra-Rexach, Alfonso J. Cruz-Jentoft

**Affiliations:** 1 Servicio de Geriatría, Hospital Universitario Puerta de Hierro de Majadahonda, 28222 Madrid, Spain; paula.sobrini@salud.madrid.org; 2 Servicio de Geriatría, Hospital Universitario Ramón y Cajal, IRYCIS, 28034 Madrid, Spain; 3 Servicio de Hematología, Hospital Universitario Ramón y Cajal, IRYCIS, 28034 Madrid, Spain; 4 Servicio de Geriatría, Hospital General Universitario Gregorio Marañón, 28007 Madrid, Spain; 5 Institute for Health Research of the Hospital General Universitario Gregorio Marañón, 28007 Madrid, Spain; 6 Biomedical Research Networking Centre on Frailty and Healthy Ageing, CIBERFES, 28029 Madrid, Spain; 7 Department of Medicine, School of Medicine, Universidad Complutense, 28040 Madrid, Spain

**Keywords:** multiple myeloma, comprehensive geriatric assessment, frailty, clinical outcomes

## Abstract

**Background/Objectives**: Multiple myeloma (MM) predominantly affects older adults, a heterogeneous population. Comprehensive Geriatric Assessment (CGA) and abbreviated tools support individualized treatment planning. This study aimed to evaluate the feasibility of CGA and its impact on clinical outcomes in this subgroup of patients. **Methods**: Prospective, observational, and single-center study including patients aged ≥65 years assessed at MM diagnosis and one year later in an onco-hematogeriatrics clinic. A CGA was performed, and frailty was evaluated using the following: G8, Geriatric Assessment in Hematology (GAH), Clinical Frailty Scale (CFS), Frail-VIG Index (FI-VIG), modified Fried phenotype, International Myeloma Working Group Frailty Index (IMWG-FI), and Revised Myeloma Comorbidity Index (R-MCI). At one year, patients were reassessed; treatment response (IMWG criteria), adverse events (CTCAE v5.03), and mortality were recorded. **Results**: Fifty-five patients (mean age 78.0 ± 5.4 years, 58.2% female) diagnosed between December 2019 and May 2024 were included. CGA completion exceeded 90% at both time points. At one year, 9 patients (16.4%) had died, and 16 (29.1%) achieved complete response, with daratumumab-lenalidomide-dexamethasone (DRd) being the most used regimen. Frailty prevalence significantly declined (FRAIL: *p* = 0.012; CFS: *p* = 0.016; IMWG-FI: *p* = 0.020). GAH was significantly associated with ≥grade 3 hematologic toxicity (OR = 5.67, *p* = 0.004) and mortality (AUC = 0.750, *p* = 0.027). FI-VIG also predicted mortality (OR = 14.67, *p* = 0.026). **Conclusions**: CGA and its abbreviated forms are feasible and clinically valuable tools for identifying vulnerable older adults with MM, enabling treatment individualization and improved risk stratification.

## Simple Summary

This study explored the use of Comprehensive Geriatric Assessment (CGA) in older adults newly diagnosed with multiple myeloma at an academic hospital in Madrid, Spain. A total of 55 patients were assessed at diagnosis, and one year later. Geriatric syndromes—including malnutrition, polypharmacy, and frailty—were highly prevalent. Although frailty status varied depending on the tool used, significant improvements were observed after one year of treatment. Frail patients were at increased risk for both grade 3–5 hematologic toxicity and mortality. These findings support the feasibility and clinical value of integrated onco-hematogeriatric care models, which facilitate multidimensional assessment and help tailor treatment strategies for vulnerable patients.

## 1. Introduction

As the population ages and emerging therapies progressively turn cancer into a chronic disease, and the growing burden of cancer in older adults constitutes a major challenge for healthcare systems. Multiple myeloma (MM) accounts for approximately 1–2% of all cancers and 17% of hematologic malignancies, making it the second most common hematologic neoplasm [1]. The median age at diagnosis is 69 years; approximately 63% of patients are over 65 years old, and up to 10% are older than 85 years [2].

Older adults with cancer are highly heterogeneous, including robust and independent individuals, as well as those who are frail or cognitively impaired. Chronological age alone is not a reliable predictor of survival, functional status, or treatment tolerance [3], and personal values and treatment preferences also vary [4].

In this context, comprehensive geriatric assessment (CGA) has emerged as a systematic, multidisciplinary tool that assesses medical, psychosocial, and functional domains, enabling the development of an individualized treatment plan and long-term follow-up [5,6]. Coordinated care models between oncologists, hematologists, and geriatricians—commonly referred to as onco-hematogeriatrics—facilitate the implementation of CGA and help stratify patients according to their risk of disability. Several tools employed by hematologists assess some of these domains. Among the tools used in MM, the International Myeloma Working Group Frailty Index (IMWG-FI) is one of the most widely used in clinical trials [7,8].

Although CGA is considered the gold standard and its use is recommended by several scientific societies [9,10,11], abbreviated approaches have also been proposed, such as the Geriatric Assessment in Hematology (GAH) [12], or a modified CGA focusing on selected domains, with a full CGA performed if deficits are identified [13]. CGA helps identify both resilient patients who can tolerate and recover from aggressive treatments and those at high risk for adverse events [14]. Numerous studies have demonstrated its prognostic value for functional decline, disability, toxicity risk, and overall survival [15,16], as well as associations with reduced postoperative complications and unplanned hospitalizations, improved treatment adherence, and enhanced quality of life [17,18,19].

However, further real-world evidence is needed in older adults with MM. Existing studies often assess only selected CGA domains or rely on frailty tools based primarily on chronological age [20,21]. Therefore, the objective of this study is to determine the feasibility and clinical utility of incorporating CGA into standard care pathways through structured collaboration between geriatricians and hematologists, evaluating its influence on patient outcomes. In parallel, the study aims to explore the evolution of frailty over time and its association with key clinical outcomes, including treatment-related toxicity, response, and survival.

## 2. Methods

### 2.1. Study Design

A prospective, single-center observational study was performed at Hospital Universitario Ramón y Cajal, Spain. The study was approved by the Independent Research Ethics Committee (ref. 225–19) and conducted by the principles of the Declaration of Helsinki. All participants provided written informed consent.

### 2.2. Inclusion and Exclusion Criteria

**Figure 1:**
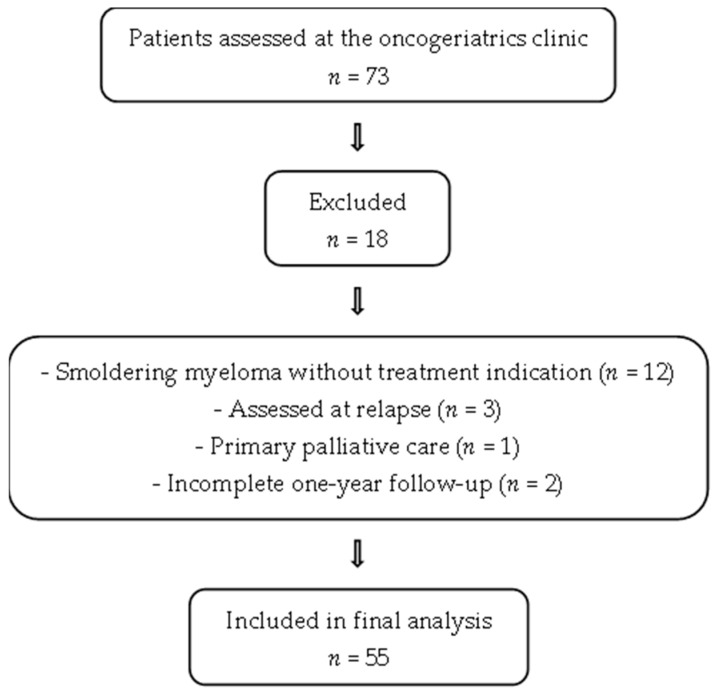
Patient Selection Flowchart.

Patients aged 65 years or older with newly diagnosed multiple myeloma (MM), according to the International Myeloma Working Group (IMWG) criteria [22], were eligible for inclusion between December 2019 and May 2024. Diagnoses were established in the hematology department, either through outpatient consultations or via referrals from the bone marrow diagnostic unit. During this period, 73 patients were assessed at the oncogeriatrics clinic at the time of diagnosis. Eighteen patients were excluded: twelve had smoldering myeloma without treatment indication, three were assessed at relapse, one was receiving primary palliative care, and two had incomplete one-year follow-up. Ultimately, 55 patients were included in the final analysis. See Figure 1.

### 2.3. Data Collection

Baseline patient characteristics—including age, sex, and comorbidities (Charlson Comorbidity Index (CCI) [23])—were extracted from medical records. Cancer-related information included the date of diagnosis and disease stage, as defined by the Revised International Staging System (R-ISS) [24].

All patients included in the study were referred to an oncogeriatric consultation. Weight and height were measured, and body mass index (BMI) was calculated. A Comprehensive Geriatric Assessment (CGA) was performed. The CGA included the Barthel Index [25] (0 = total dependence, 100 = full independence) and Lawton-Brody Index (0 = dependence, 8 = instrumental independence), for functional status [26]; the Mini-Mental State Examination (MMSE) [27] and the Geriatric Depression Scale (GDS) [28] by Yesavage for cognitive and emotional status; and the Short Form of the Mini Nutritional Assessment (MNA-SF) [29] for nutritional evaluation. From a social perspective, it was recorded whether patients lived alone, with family, or in a residential care facility.

Geriatric syndromes were assessed, including malnutrition (based on the Global Leadership Initiative on Malnutrition (GLIM) criteria [29]), falls (number of falls in the past year [30]), and polypharmacy [31], defined as the use of five or more medications, including eye drops, laxatives, and over-the-counter drugs. According to the deficits detected in the CGA, supportive interventions were systematically implemented. Most patients received nutritional counseling with recommendations to enrich protein intake. Those meeting GLIM criteria for malnutrition were prescribed oral dietary supplements, predominantly hyperprotein and hypercaloric, twice daily for three months [32]. In addition, all patients were encouraged to engage in multicomponent physical exercise as part of their routine supportive care [33].

Frailty was evaluated using the FRAIL scale (score ≥ 1 indicating frailty risk), as well as the G8 [34] screening tool (score ≤ 14 indicating frailty risk), and the Geriatric Assessment in Hematology (GAH) [13] score (≥42 indicating frailty risk). The Frail-VIG index [35] (VIG is the Spanish abbreviation for Comprehensive Geriatric Assessment) was also calculated (<0.20: robust; 0.20–0.35: mildly frail; 0.35–0.55: moderately frail; 0.55–0.70: severely frail). Frailty was further confirmed using the Clinical Frailty Scale (CFS) by Rockwood (9-point pictorial scale, with scores ≥ 4 indicating frailty) and the modified Fried phenotype criteria, which assess five components (weight loss, exhaustion, physical inactivity, low grip strength, and slowness); the presence of three or more components defines frailty [36], and this tool is considered the reference standard for physical frailty. These results were compared with the International Myeloma Working Group Frailty Index (IMWG-FI) (0: robust, 1: pre-frail, ≥2: frail) and the Revised Myeloma Comorbidity Index (R-MCI) (≤3: robust, 4–6: pre-frail, >6: frail). In both assessment tools, age ≥ 75 years contributes 1 or 2 points to the total score.

Muscle strength was assessed following the revised European Working Group on Sarcopenia in Older People (EWGSOP2) [37] definition, by measuring handgrip strength using a Jamar hydraulic dynamometer under standardized conditions. Probable sarcopenia was defined as handgrip strength < 16 kg in women and < 27 kg in men. Physical performance was evaluated by the 4-meter gait speed test; a walking speed ≤ 0.8 m/s was considered low.

The feasibility of CGA was evaluated based on completion rates and data availability at two points: baseline and follow-up. Baseline was defined as the time of diagnosis, before the start of treatment or within the first cycle. Follow-up was performed one year after the baseline assessment, corresponding to approximately one year after treatment initiation. A CGA was considered complete for a given patient if at least 80% of the assessment domains were filled and key domains: functional status (Barthel Index), cognition (MMSE), nutrition (GLIM criteria), and frailty (Fried or Rockwood, as in some cases physical performance test could not be performed, mostly at diagnosis) were not missing.

Patients were treated according to the protocols of the hematology department; the assessment was not intended to interfere with or modify the established therapeutic regimen.

### 2.4. Follow-Up and Assessment of Frailty Evolution

Medical records of all included patients were reviewed to collect information on treatment regimens, adverse events, best treatment response, and mortality. One year after the initial onco-hematogeriatric consultation, surviving patients were re-evaluated in the clinic, and the same geriatric assessment tools used at baseline were reapplied. Treatment response was documented according to the 2016 International Myeloma Working Group (IMWG) criteria [38], which include conventional response categories—such as stringent complete response, complete response, very good partial response, partial response, minimal response, stable disease, and progressive disease—based on biochemical, bone marrow, and imaging parameters. In addition, minimal residual disease (MRD) negativity, assessed by flow cytometry or next-generation sequencing combined with PET-CT, was recorded as the deepest response level. Adverse events were registered according to the Common Terminology Criteria for Adverse Events version 5.03 (CTCAE) [39], with a specific focus on grade 3–5 hematologic toxicities. However, due to incomplete documentation in a significant number of cases, the severity of non-hematologic toxicities could not be assessed and was, therefore, excluded from statistical analysis. Reported non-hematologic toxicities included atrial fibrillation, heart failure, nausea, fatigue, renal impairment, infections, and peripheral neuropathy, among others.

To explore the dynamic nature of frailty, frailty status at one year was compared to baseline using the main assessment tools (G8, GAH, FRAIL, modified Fried, CFS, FI-VIG, IMWG-FI, and R-MCI). Patients were classified according to frailty trajectory as improved, stable, or worsened. The proportion of patients in each category was analyzed to better understand functional evolution in this population.

One year was considered an adequate timeframe to evaluate the association between frailty, CGA components, and key clinical outcomes (toxicity, treatment response, and mortality), given that most adverse events and resistance typically occur within the first year of treatment. Continuous reassessment was not feasible within the study scope.

### 2.5. Statistical Analyses

Continuous variables were expressed as means and standard deviations or as medians and interquartile ranges, depending on their distribution. Categorical variables were presented as absolute values and percentages. The independent t-test or Mann–Whitney U test was used to compare continuous variables, while the χ^2^ test or Fisher’s exact test was applied for categorical variables.

To evaluate changes in frailty, functional status, and other geriatric domains between baseline and one-year follow-up, paired analyses were performed. The choice of statistical test was based on the nature of each variable: the Wilcoxon signed-rank test was used for continuous or ordinal non-normally distributed variables; the paired Student’s t-test was applied when normality was assumed. McNemar’s test was used to assess differences in dichotomous paired variables, while the Friedman test was applied to ordinal variables with more than two categories, such as frailty classifications.

Associations between CGA components and treatment-related outcomes (toxicity and mortality) were analyzed using multivariable logistic regression, with odds ratios (ORs) and 95% confidence intervals (CIs) reported. In a first step, CGA domains (functional status, cognition, nutrition, frailty, comorbidity, mood, polypharmacy, falls, and social situation) were included to identify which components were independently associated with each outcome. In a second step, simplified geriatric scores were evaluated in separate models to compare their predictive performance with the full CGA. Discrimination was assessed using receiver operating characteristic (ROC) curve analysis and area under the curve (AUC) values.

Overall survival (OS) and progression-free survival (PFS) were estimated using Kaplan–Meier curves and compared using the log-rank test.

All *p*-values were two-sided, with statistical significance set at 0.05. Missing data were limited to a few baseline assessments, mostly involving 1–2 items per patient. Sarcopenia data were missing in 11 cases due to difficulties obtaining handgrip measurements early in the study. No imputation was performed, and analyses were based on available cases. Statistical analyses were performed using SPSS software, version 25.0 for Windows (IBM Corp., Armonk, NY, USA).

## 3. Results

### 3.1. Study Population, Baseline Characteristics, and CGA Completion

A total of 55 patients with newly diagnosed multiple myeloma (MM) were included between December 2019 and May 2024. The mean age was 78.0 ± 5.4 years, with 47% aged 80 years or older, and 58.2% were female. The average Charlson Comorbidity Index (CCI) was 3.0 ± 2.0. Most patients presented with R-ISS stage II disease (65.5%).

Over 90% of patients completed the Comprehensive Geriatric Assessment (CGA) after referral to the onco-hematogeriatric clinic, both at baseline and at one-year follow-up among survivors, with full follow-up and no statistically significant differences observed (Table 1). Baseline characteristics are detailed in Table 2. Approximately 50% were independent in basic and instrumental activities of daily living (49.1% Barthel = 100, 41.8% Lawton = 8), with no cognitive impairment or depressive symptoms. However, geriatric syndromes were prevalent: 85.5% had polypharmacy, and a high proportion were malnourished per GLIM criteria and were likely sarcopenic with poor physical performance.

Frailty was highly prevalent but showed substantial variation depending on the assessment tool used. According to the modified Fried criteria—considered the reference standard for physical frailty—47.3% of patients were classified as frail. The Clinical Frailty Scale (CFS) identified 56.4% as frail, while the IMWG-FI and FI-VIG classified 65.5% and 58.2%, respectively. The R-MCI yielded a lower estimate (12.7%). Notably, the GAH—a screening tool specifically developed for hematologic patients—identified 54.5% as frail, a figure very close to that of the modified Fried criteria, suggesting good alignment with the reference standard. In contrast, other screening tools such as the G8 (89.1%) and FRAIL (83.6%) produced substantially higher prevalence rates, likely reflecting their greater sensitivity.

Treatment regimens were guided by hematology protocols: daratumumab, lenalidomide, and dexamethasone (DRd) were most common (21 patients, 38.2%), followed by daratumumab, bortezomib, melphalan, and prednisone (D-VMP; 16 patients, 29.1%), lenalidomide and dexamethasone (Rd; 6, 10.9%), clinical trial GEM2017Fit [40] (5, 9.1%), and various other regimens (13.6%). Starting in 2021, and in accordance with clinical practice guideline recommendations [41], frailty screening tools such as the G8 and the GAH began to be applied in our hematology department. These assessments mainly influenced the dosing of certain drugs, particularly corticosteroids and lenalidomide.

### 3.2. One-Year Follow-Up and Functional Changes

**Figure 2:**
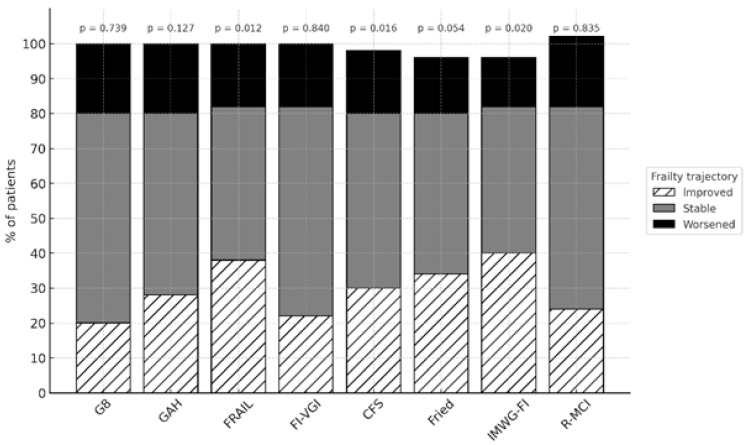
Frailty trajectories from baseline to one-year follow-up according to different frailty tools. Bars indicate the percentage of patients whose frailty status improved, remained stable, or worsened. Denominators (n) for each scale are: G8 (*n* = 54), GAH (*n* = 54), FRAIL (*n* = 54), FI-VIG (*n* = 55), CFS (*n* = 55), Fried (*n* = 49), IMWG-FI (*n* = 55), and R-MCI (*n* = 55).

During the one-year follow-up, nine patients (16.4%) died, one of whom before treatment initiation. These cases were excluded from paired comparisons due to a lack of follow-up data. Baseline characteristics at one year for the 46 survivors are summarized in Table 2. Statistically significant improvements were observed in nutritional status, with malnutrition decreasing from 47% to 21% (*p* = 0.041), and in frailty risk measured by FRAIL (83.6% to 58.7%, *p* = 0.031). No significant differences were found in functional or cognitive status. Although there was an increase in patients with low muscle strength (56.4% to 63.0%), the change was not statistically significant. Frailty trajectory changes from baseline to one year are shown in Figure 2. Statistically significant improvements in frailty status over one year were observed in the FRAIL (*p* = 0.012), CFS (*p* = 0.016), and IMWG-FI (*p* = 0.020) scales, with a reduction in the number of patients classified as frail and a corresponding shift toward pre-frail or robust categories.

### 3.3. Clinical Outcomes: Toxicity, Response, and Survival

At one year, 16 patients (29.1%) achieved complete response (CR). Additionally, 12 (21.8%) reached partial response (PR), 16 very good partial response (VGPR), 5 (10.9%) had stable disease, 2 (3.6%) patients showed disease progression, and 4 (7.3%) were not evaluable due to early death. Adverse events occurred in 61.8% of patients and led to treatment discontinuation in 14 cases (41.1%). Hematological toxicity grade 3–5 affected 29 patients (52.7%), and 51 experienced non-hematological toxicity, with asthenia being the most common (52.7%). Table 3 summarizes mortality, treatment response, and grade ≥3 hematological toxicity by frailty level. Statistically significant associations were found between frailty risk by GAH and both mortality (*p* = 0.033) and grade >3 hematological toxicity (*p* = 0.003). FRAIL ≥ 1 and CFS ≥ 4, were associated with grade 3–5 hematological toxicity (*p* = 0.020 and *p* = 0.047, respectively). Additionally, FI-VIG was associated with mortality (*p* = 0.038).

Multivariable logistic regression analyses were conducted to evaluate the association between CGA components and clinical outcomes. See Tables S1–S3. Although most variables did not yield clinically significant results, the Charlson Comorbidity Index showed strong discriminative ability for predicting mortality (AUC = 0.835, *p* = 0.0075). Among frailty scales, only FI-VIG was significantly associated with mortality: frail patients had a higher likelihood of death (OR = 14.67, 95% CI: 1.37–156.89, *p* = 0.026). For grade 3–5 hematological toxicity, GAH (OR = 5.67, 95% CI: 1.75–18.38, *p* = 0.004) and FRAIL (OR = 10.32, 95% CI: 1.17–90.78, *p* = 0.035) were significantly associated with increased risk. No frailty scale showed a statistically significant association with treatment response. See Tables S4–S6. However, odds ratios were generally <1, indicating a possible trend toward lower response rates in frailer individuals, although these findings were not statistically significant (*p* > 0.05).

Among frailty scales, GAH (AUC = 0.750, *p* = 0.027), FI-VIG (AUC = 0.747, *p* = 0.029), and Modified Fried (AUC = 0.719, *p* = 0.053) showed acceptable discriminative ability for predicting mortality.

**Figure 3:**
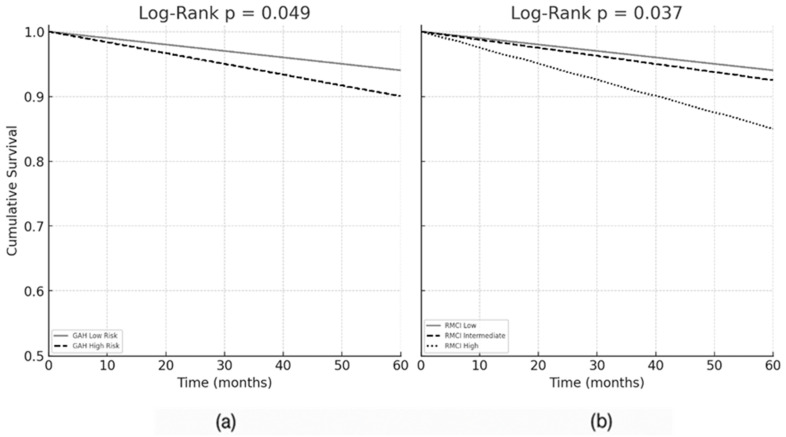
Kaplan–Meier survival curves. (**a**) Overall survival according to the Geriatric Assessment in Hematology (GAH) scale: low-risk (*n* = 24) vs. high-risk (*n* = 30). (**b**) Overall survival according to the Revised Myeloma Comorbidity Index (R-MCI): low (*n* = 12), intermediate (*n* = 36), and high (*n* = 7) risk groups. Log-rank p-values are shown.

The estimated mean overall survival (OS) was 47.9 months (95% CI: 43.3–52.4). Statistically significant OS differences were found only for GAH and R-MCI scales (log-rank *p* = 0.049 and *p* = 0.037, respectively), as shown in Figure 3. Other scales (Fried, FRAIL, IMWG-FI, FI-VIG, G8, and CFS) showed non-significant trends towards shorter survival among frail patients.

The estimated mean progression-free survival (PFS) was 42.2 months (95% CI: 36.4–48.0). No frailty scale showed statistically significant differences in PFS across groups (log-rank *p* > 0.05), although lower PFS was noted in more frail categories, particularly in FI-VIG (21.4 months) and Modified Fried (41.1 months).

## 4. Discussion

This prospective study assessed the feasibility and clinical relevance of Comprehensive Geriatric Assessment (CGA) and frailty measures in a real-world cohort of older adults with newly diagnosed multiple myeloma. Recognizing the complexity of managing older cancer patients and the numerous applications of geriatric assessment, the field of geriatric hematology—or hematogeriatrics—is increasingly gaining ground in clinical practice [17,42,43]. This study demonstrates the feasibility of maintaining close and coordinated collaboration between geriatrics and hematology, achieving CGA completion rates above 90% not only at diagnosis but also at one-year follow-up. Other published studies have also demonstrated the feasibility of CGA, although most have applied only partial assessments focused on selected domains [20].

The deficits detected by a CGA evolve throughout the disease course, particularly frailty. In 2023, Mian et al. [44] demonstrated the evolution of frailty status in patients with multiple myeloma—both improvement and deterioration—highlighting the importance of reassessing frailty to guide treatment adjustments. In our study, significant changes were observed one year after diagnosis and treatment initiation, particularly in nutritional status and frailty. These improvements may partly reflect the effect of supportive interventions systematically recommended according to CGA deficits, including nutritional counseling, prescription of hypercaloric and hyperprotein oral supplements in patients meeting GLIM criteria for malnutrition, and encouragement of multicomponent physical exercise. A statistically significant improvement in frailty status was found using the FRAIL, CFS, and IMWG-FI tools. These findings underscore the importance of identifying when frailty is primarily disease-related and potentially reversible with appropriate therapy.

Additionally, we observed a heterogeneous population in terms of frailty presence and severity, with wide variation depending on the assessment tool used, a finding also reported in the MFRAIL study by Haider et al. [45] In our cohort, the proportion of patients classified as frail ranged from nearly 90% with the G8 to less than half with the Fried phenotype, and only 12.7% with the R-MCI, while the GAH identified about 55%. This discordance has direct clinical implications, as treatment intensity could vary substantially depending on the tool applied. Screening instruments such as the G8 maximize sensitivity but may overestimate frailty, leading to potential undertreatment, whereas tools focused mainly on physical performance (e.g., Fried) may miss deficits in other domains, and comorbidity-based tools like the R-MCI may underestimate frailty in patients with significant functional or nutritional impairment. Some tools may misclassify patients, labeling those with established disability and palliative care needs as frail, while truly frail but potentially recoverable individuals are classified as pre-frail. For this reason, CGA is considered the reference standard for evaluating frailty in older adults. The combination of assessed domains—such as polypharmacy, depression, sarcopenia, and others—provides the necessary information to develop individualized care plans and optimize treatment strategies [18,46].

This study identified a clear association between frailty and clinical outcomes. Specifically, frailty risk measured by the GAH scale was significantly associated with mortality and grade ≥3 hematologic toxicity. Logistic regression confirmed that higher GAH scores independently predicted increased toxicity risk, and ROC analysis showed it had acceptable accuracy in predicting mortality. The GAH scale was specifically developed for patients with hematologic malignancies [12], is sensitive to clinical changes [47], and can discriminate patients at risk of treatment-related toxicity. Despite the limited sample size, the study reinforces the GAH’s value in identifying vulnerable patients and supporting personalized treatment decisions in daily clinical practice.

On the other hand, the results obtained with the FI-VIG are also noteworthy. This frailty index, based on the deficit accumulation model, shares the multidimensional nature of the CGA [48]. In our study, FI-VIG was significantly associated with mortality, with higher scores corresponding to increased risk, and it showed acceptable discriminative ability in ROC curve analysis. This tool has previously demonstrated prognostic value in various clinical settings [49] and across different patient populations [50], but it had not yet been evaluated in older adults with hematologic malignancies, specifically multiple myeloma. Therefore, our findings support the potential utility of FI-VIG in the field of geriatric hematology.

In contrast, no statistically significant associations were observed for the IMWG-FI scale, despite it being one of the most widely used tools in clinical trials involving patients with MM [7]. This score combines age, ADL, IADL, and CCI [51]. In this line, Muzyka et al. [52] demonstrated that the 40-item Rockwood FI was significantly associated with OS, showing non-inferiority to the IMWG-FI. From a geriatric perspective, it is important to recognize that frailty is a process independent of chronological aging, which may reduce the discriminative capacity of age-based tools in older populations [53]. As shown in our study, the number of comorbidities strongly predicts mortality, yet it may be the multidimensional nature of the evaluation that provides greater clinical relevance.

It is also worth noting that, given the high prevalence of frailty in our cohort, the overall survival (OS) was 47.9 months and the PFS was 42.2 months, which is lower than that reported in clinical trials involving fitter patients [7,54]. This discrepancy is largely explained by older age and higher prevalence of frailty and comorbidities in our cohort, which more closely resembles real-world practice. Indeed, observational studies in unselected older patients with MM have reported median OS values in the range of 30–50 months and substantially shorter PFS compared to trial populations, which is consistent with our findings [54,55]. These findings point to the need for implementing coordinated care models between geriatrics and hematology that ensure thorough, multidimensional evaluation of patients and stress the significance of clinical context and personalized judgment.

Several limitations should be acknowledged. First, the relatively small sample size (*n* = 55) limits the statistical power to detect small-to-moderate effects and precludes definitive conclusions about prognostic utility. Confidence intervals were wide in several models, and certain associations may have been under- or over-estimated. As a result, the findings should be interpreted with caution and considered exploratory until validated in larger cohorts. In addition, treatment was not modified based on CGA results, so its direct impact on therapeutic decision-making could not be evaluated. This represents an important limitation, as the integration of CGA findings into treatment decisions is essential to maximize its clinical value. Nevertheless, our findings highlight the potential clinical utility of CGA and frailty tools to guide treatment strategies in daily practice. A major strength of this study lies in its prospective design and real-world setting, which enhances the clinical applicability of the findings. Furthermore, the use of multiple frailty tools and a complete CGA allowed for a nuanced analysis of geriatric vulnerability and its evolution over time. At present, it is unlikely that a single “one-size-fits-all” tool could fully capture the multidimensional nature of frailty in older adults with MM. While simplified models may facilitate screening, the CGA remains the reference standard for tailoring treatment decisions.

## 5. Conclusions

Geriatric onco-hematology models are feasible and ensure a multidimensional evaluation of patients with hematologic malignancies—particularly multiple myeloma—through the use of Comprehensive Geriatric Assessment (CGA). Abbreviated tools such as the GAH scale and the FI-VIG index have shown clinical value by facilitating the early identification of patients at higher risk for treatment-related toxicity or mortality, thereby informing tailored interventions. Given the increasing number of older adults diagnosed with cancer, particularly multiple myeloma, coordinated and collaborative care models are essential. Their incorporation into routine hematology practice could help personalize treatment strategies and improve outcomes in this vulnerable population. Future studies with larger cohorts are warranted to determine which frailty assessment system best predicts outcomes and supports clinicians in selecting the most appropriate tool for their patients.

## Figures and Tables

**Table 1:** CGA completion.

Indicator	Baseline *n* = 55 (%)	1-Year *n* = 46 (%)	*p*-Value
CGA Domain Completion (Threshold ≥80%) ^1^	53 (96.4)	45 (97.8)	0.448
Functional status present	55 (100.0)	46 (100.0)	NA
Cognition present	54 (98.2)	45 (97.8)	NA
Nutrition present	55 (100.0)	46 (100.0)	NA
Frailty Fried Rockwood	49 (89.1) 54 (98.2)	46 (100.0) 46 (100.0)	NA
Complete CGA (≥80% + key items ^2^)	53 (96.4)	45 (97.5)	0.333

^1^ A complete Comprehensive Geriatric Assessment (CGA) includes the following domains: comorbidity (evaluated using the Charlson Comorbidity Index), functional status (Barthel Index and Lawton Scale), cognitive function (MMSE), mood (Yesavage Geriatric Depression Scale), nutritional status (GLIM criteria), gait speed, muscle strength, frailty (assessed by both Fried phenotype and Rockwood Clinical Frailty Scale), polypharmacy, and history of falls and social situation. ^2^ Functional status (Barthel), cognition (MMSE), nutrition (GLIM), and frailty (Fried or Rockwood) were considered key components of the CGA.

**Table 2:** Baseline characteristics at diagnosis and one year later.

Variable	At Diagnosis (*n* = 55) ^1^	One Year Later (*n* = 46) ^1^	*p* Value ^2^
Basic activities of daily living (Barthel)	90.4 ± 14.6	89.0 ± 19.4	0.126
Instrumental activities of daily living (Lawton)	5.4 ± 2.8	5.7 ± 2.5	0.759
Mental status (MMSE)	27.3 ± 3.1	26.8 ± 3.7	0.111
Depression (GDS) ≥6 (suspected depression) NI	18 (32.0) –	15 (32.6) 2 (4.3)	1.00
Living arrangement Lives alone Lives with family Institutionalized	14 (25.5) 40 (72.7) 0 (0.0)	8 (17.4) 35 (76.1) 3 (6.5)	
Nutritional status BMI (kg/m^2^) MNA-SF: ≤11 (malnutrition risk) GLIM criteria (yes)	26.2 ± 4.2 39 (70.9) 26 (47.3)	26.8 ± 4.2 14 (30.4) 10 (21.7)	0.846 0.003 0.041
Falls (≥1 in the last year)	13 (23.6)	6 (13.0)	0.832
Polypharmacy (≥5)	47 (85.5)	42 (91.3)	0.219
Frailty FRAIL (≥1) NI G8 (≤14) NI GAH (>42) NI FI-VIG <0.2 (robust) 0.2–0.35 (mild frail) 0.35–0.7 (moderate-severe frail) CFS 1–3 (robust) ≥4 (frail) Modified Fried 0 (robust) 1–2 (pre-frail) ≥3 (frail) NI IMWG-FI 0 (robust) 1 (pre-frail) ≥2 (frail) R-MCI ≤3 (robust) 4–6 (pre-frail) >6 (frail)	46 (83.6) 1 (1.8) 49 (89.1) 1 (1.8) 30 (54.5) 1 (1.8) 23 (41.8) 22 (40.0) 10 (18.2) 24 (43.6) 31 (56.4) 4 (7.3) 19 (34.5) 26 (47.3) 6 (10.9) 2 (3.6) 17 (30.9) 36 (65.5) 12 (21.8) 36 (65.6) 7 (12.7)	27 (58.7) – 40 (87.0) – 15 (32.6) – 23 (50.0) 15 (32.6) 8 (17.4) 34 (73.9) 12 (26.1) 10 (21.7) 20 (43.5) 16 (38.8) – 2 (4.3) 8 (17.4) 36 (78.3) 13 (28.3) 29 (63.0) 4 (8.7)	0.031 1.00 0.189 0.840 0.052 0.054 0.020 0.835
Probable sarcopenia (muscle strength [kg]) NI	31 (56.4) 11 (20.0)	29 (63.0) –	1.00
Physical performance Slow gait speed (≤0.8 m/s) NI	32 (58.2) 1 (1.8)	20 (43.5) –	0.167

^1^ *n* (%), for qualitative variables; mean ± standard deviation, for quantitative. ^2^ Continuous variables were compared using the paired Student’s t-test or the Wilcoxon signed-rank test, depending on data distribution. Categorical variables with two paired categories (e.g., yes/no) were analyzed using McNemar’s test. A two-sided *p*-value < 0.05 was considered statistically significant. MMSE, Mini-Mental State Examination; GDS, Yesavage’s Geriatric Depression Scale; BMI, Body Mass Index, MNA-SF, Mini Nutritional Assessment Short Form; GLIM, Global Leadership Initiative on Malnutrition; G8, Geriatric 8 scale; GAH, Geriatric Assessment in Hematology scale; Frail-VIG Index, VIG is the Spanish abbreviation for Comprehensive Geriatric Assessment; CFS, Rockwood’s Clinical Frailty Scale; IMWG-FI, International Myeloma Working Group Frailty Index; R-MCI, Revised Myeloma Comorbidity Index; NA, not applicable; NI, no information for these patients was collected.

**Table 3:** Impact of frailty on clinical treatment outcomes.

Frailty Scale (Criteria)	Mortality *n* (%) ^1^ *p*-Value ^2^	Hematological Toxicity ≥ G3 *n* (%) *p*-Value	Response ≥ VGPR ^3^ *n* (%) *p*-Value
G8 (≤14), *n* ^4^ = 49	9 (18.4) 0.576	27 (55.1) 0.184	25 (55.6) 1.00
GAH (>42), *n* = 30	8 (26.7) 0.033	21 (70.0) 0.003	13 (48.1) 0.226
FRAIL (≥1), *n* = 46	9 (19.1) 0.176	28 (59.6) 0.020	24 (55.8) 0.762
FI-VIG <0.2 (robust), *n* = 23 0.2–0.35 (mild frail), *n* = 22 0.35–0.7 (moderate–severe frail), *n* = 10	1 (4.3) 4 (18.2) 4 (40.0) 0.038	9 (39.1) 13 (59.1) 7 (70.0) 0.196	13 (59.1) 11 (55.0) 4 (44.4) 0.758
CFS 1–3 (robust), *n* = 24 ≥4 (frail), *n* = 31	1 (4.2) 8 (25.8) 0.062	9 (37.5) 20 (64.5) 0.047	12 (50.0) 16 (59.3) 0.507
Modified Fried 0 (robust), *n* = 4 1–2 (pre-frail), *n* = 19 ≥3 (frail), *n* = 26	0 (0.0) 1 (5.3) 7 (26.9) 0.099	1 (25.0) 10 (52.6) 15 (57.7) 0.475	3 (75.0) 12 (63.2) 10 (43.5) 0.305
IMWG-FI 0 (robust), *n* = 2 1 (pre-frail), *n* = 17 ≥2 = Frail, *n* = 36	0 (0.0) 0 (0.0) 9 (25.0) 0.058	1 (50.0) 7 (41.2) 21 (58.3) 0.504	2 (100.0) 8 (47.1) 18 (56.3) 0.352
R-MCI ≤3 (robust), *n* = 12 4–6 (pre-frail), *n* = 36 >6 (frail), *n* = 7	0 (0.0) 6 (16.7) 3 (42.9) 0.051	4 (33.3) 22 (61.1) 3 (42.9) 0.212	9 (75.0) 17 (51.5) 2 (33.3) 0.198

^1^ Percentages are calculated within each frailty category (row-wise). ^2^ Chi-square or Fisher’s exact test was used as appropriate; Fisher’s test was applied when expected cell counts were <5. ^3^ VGPR, Very Good Partial Response. ≥VGPR encompasses complete response and stringent complete response. ^4^ Denominators (*n*) for each frailty category are shown in the first column and correspond to the baseline distribution presented in Table 1. G8, Geriatric 8 scale; GAH, Geriatric Assessment in Hematology scale; Frail-VIG Index, VIG is the Spanish abbreviation for Comprehensive Geriatric Assessment; CFS, Rockwood’s Clinical Frailty Scale; IMWG-IF, International Myeloma Working Group Frailty Index; R-MCI, Revised Myeloma Comorbidity Index.

## Data Availability

The datasets generated and/or analyzed during the current study are available from the corresponding author upon reasonable request.

## Supplementary Materials

The following supporting information can be downloaded at the following address: https://www.mdpi.com/article/10.3390/cancers17172904/s1, Table S1: Logistic regression. CGA and mortality; Table S2: Logistic regression. CGA and Hematological Toxicity (Grades 3–5); Table S3: Logistic regression. CGA and treatment response; Table S4: Logistic regression. Frailty and mortality; Table S5: Logistic regression. Frailty and hematological G3–5 toxicity; Table S6: Logistic regression. Frailty and treatment response.

## Abbreviations

The following abbreviations are used in this manuscript:

MM	Multiple Myeloma
CGA	Comprehensive Geriatric Assessment
ADL	Activities of Daily Living
IADL	Instrumental Activities of Daily Living
IMWG	International Myeloma Working Group
IMWG-FI	International Myeloma Working Group Frailty Index
GAH	Geriatric Assessment in Hematology
FI-VIG	Frailty Index—Valoración Integral Geriátrica
CFS	Clinical Frailty Scale
R-MCI	Revised Myeloma Comorbidity Index
GLIM	Global Leadership Initiative on Malnutrition
BMI	Body Mass Index
MMSE	Mini-Mental State Examination
GDS	Geriatric Depression Scale
MNA-SF	Mini Nutritional Assessment—Short Form
EWGSOP2	European Working Group on Sarcopenia in Older People (2nd ed.)
CTCAE	Common Terminology Criteria for Adverse Events
MRD	Minimal Residual Disease
PET-CT	Positron Emission Tomography—Computed Tomography
OR	Odds Ratio
CI	Confidence Interval
ROC	Receiver Operating Characteristic
AUC	Area Under the Curve
OS	Overall Survival
PFS	Progression-Free Survival
CR	Complete Response
VGPR	Very Good Partial Response
PR	Partial Response
DRd	Daratumumab, Lenalidomide, Dexamethasone
D-VMP	Daratumumab, Bortezomib, Melphalan, Prednisone
Rd	Lenalidomide, Dexamethasone
CCI	Charlson Comorbidity Index
R-ISS	Revised International Staging System
NA	Not Applicable
NI	No Information
SPSS	Statistical Package for the Social Sciences
IBM	International Business Machines (Corp.)
