# Supplementary material for: Comprehensive Geriatric Assessment of Older Patients with Multiple Myeloma: A Prospective Observational Study
Source: Cancers (Basel). 2025 Sep 4;17(17):2904. doi: 10.3390/cancers17172904 (PMC12428207; doi:10.3390/cancers17172904)
Supplement: Supplementary file 1 [file cancers-17-02904-s001.zip › cancers-3825187-supplementary.pdf]

cancers MDPI

Supplementary Material: Comprehensive Geriatric Assessment in Older Patients with Multiple Myeloma: a Prospective Observational Study

Paula Sobrini-Morillo, Celia Corral-Tuesta, Carmen Sánchez-Castellano, Tamara Gutiérrez-Blanco, Pablo Palomo-Rumschisky, Claudia Gabriela Álvarez-Pinheiro, María Jesús Blanchard-Rodríguez, José A. Serra-Rexach and Alfonso J. Cruz-Jentoft

Table S1. Logistic regression. CGA and mortality.

Variable	Odds Ratio (OR)	95% CI (Lower – Upper)	*p*-value
CCI	3.42×10^{21}	0.00– .	0.982
Barthel	0.001	$0.00–2.36 \times 10^{284}$	0.984
MMSE	1.66×10^{11}	0.00– .	0.983
GDS	0.00	0.00– .	0.985
GLIM	3.88×10^{100}	0.00– .	0.984
Falls	0.00	0.00– .	0.984
Polypharmacy	0.00	0.00– .	0.984
Lives alone	1.29×10^{96}	0.00– .	0.985
Fried			
Pre-frail vs fit	0.00	0.00– .	0.997
Frail vs fit	1.89×10^{28}	0.00– .	0.997

CCI, Charlson Comorbidity Index; MMSE, Mini-Mental State Examination; GDS, Yesavage's Geriatric Depression Scale; GLIM, Global Leadership Initiative on Malnutrition.

Table S2. Logistic regression. CGA and Hematological Toxicity (Grades 3–5).

Variable	Odds Ratio (OR)	95% CI (Lower – Upper)	*p*-value
CCI	1.22	0.75–1.97	0.416
Barthel	1.01	0.94–1.08	0.776
MMSE	0.80	0.56–1.16	0.260
GDS	0.59	0.09–3.72	0.575
GLIM	0.99	0.21–4.67	0.992
Falls	0.76	0.07–7.35	0.816
Polypharmacy	6.59	0.75–58.07	0.089
Lives alone	2.43	0.38–15.53	0.347
Modified Fried			
Pre-frail vs fit	2.81	0.15–52.07	0.487
Frail vs fit	1.94	0.09–40.67	0.667

CCI, Charlson Comorbidity Index; MMSE, Mini-Mental State Examination; GDS, Yesavage's Geriatric Depression Scale; GLIM, Global Leadership Initiative on Malnutrition.

Table S3. Logistic regression. CGA and treatment response.

Variable	Odds Ratio (OR)	95% CI	*p*-value
CCI	0.48	0.19–1.18	0.111
Barthel	1.08	0.96–1.22	0.164
MMSE	0.85	0.52–1.38	0.513
GDS	12.01	1.22–117.35	0.033
GLIM	0.74	0.12–4.42	0.746
Falls	0.15	0.01–2.08	0.158
Polypharmacy	0.59	0.06–5.82	0.653

Lives alone	0.12	0.01–1.25	0.077
Modified Fried			
Pre-frail vs fit	0.24	0.01–5.74	0.381
Frail vs fit	0.46	0.02–10.96	0.634

CCI, Charlson Comorbidity Index; MMSE, Mini-Mental State Examination; GDS, Yesavage's Geriatric Depression Scale; GLIM, Global Leadership Initiative on Malnutrition.

Table S4. Logistic regression. Frailty and mortality.

**Frailty Scale**	**Odds Ratio (OR)**	**95% CI (Lower – Upper)**	***p*-value**
G8	3.63×10^8	0.00– .	0.999
GAH	8.36	0.97–72.48	0.054
FRAIL	3.83×10^8	0.00– .	0.999
FI-VIG			
Intermediate vs fit	4.89	0.50–47.71	0.172
Frail vs fit	14.67	1.37–156.89	0.026
CFS	8.00	0.93–69.21	0.059
Modified Fried			
Intermediate vs fit	8.97×10^7	0.00– .	0.999
Frail vs fit	5.95×10^8	0.00– .	0.999
IMWG-FI			
Intermediate vs fit	1.00	0.00– .	1.000
Frail vs fit	5.38×10^8	0.00– .	0.999
R-MCI			
Intermediate vs fit	3.23×10^8	0.00– .	0.999
Frail vs fit	1.21×10^9	0.00– .	0.999

G8, Geriatric 8 scale; GAH, Geriatric Assessment in Hematology scale; Frail-VIG Index, VIG is the Spanish abbreviation for Comprehensive Geriatric Assessment; CFS, Rockwood's Clinical Frailty Scale; IMWG-FI, International Myeloma Working Group Frailty Index; R-MCI, Revised Myeloma Comorbidity Index.

Table S5. Logistic regression. Frailty and hematological G3-5 toxicity.

**Frailty Scale**	**Odds Ratio (OR)**	**95% CI (Lower – Upper)**	**p-value**
G8	4.91	0.51–47.16	0.168
GAH	5.67	1.75–18.38	0.004
FRAIL	10.32	1.17–90.78	0.035
FI-VIG			
Intermediate vs fit	2.25	0.68–7.41	0.184
Frail vs fit	3.63	0.74–17.81	0.112
CFS	3.03	1.00–9.16	0.050
Modified Fried			
Prefrail vs fit	3.33	0.29–38.08	0.333
Frail vs fit	4.09	0.37–44.79	0.249
IMWG-FI			
Intermediate vs fit	0.70	0.04–13.18	0.812
Frail vs fit	1.40	0.08–24.20	0.817
R-MCI			
Intermediate vs fit	3.14	0.80–12.43	0.103
Frail vs fit	1.50	0.22–10.22	0.679

G8, Geriatric 8 scale; GAH, Geriatric Assessment in Hematology scale; Frail-VIG Index, VIG is the Spanish abbreviation for Comprehensive Geriatric Assessment; CFS, Rockwood's Clinical Frailty Scale; IMWG-FI, International Myeloma Working Group Frailty Index; R-MCI, Revised Myeloma Comorbidity Index.

Table S6. Logistic regression. Frailty and treatment response.

Frailty Scale	Odds Ratio (OR)	95% CI (Lower – Upper)	*p*-value
G8	0.79	0.12–5.21	0.810
GAH	0.46	0.15–1.44	0.183
FRAIL	1.20	0.27–5.42	0.813
FI-VIG			
Intermediate vs fit	0.85	0.25–2.88	0.789
Frail vs fit	0.46	0.10–2.12	0.320
CFS	1.33	0.45–3.99	0.607
Modified Fried			
Prefrail vs fit	0.57	0.05–6.61	0.654
Frail vs fit	0.24	0.02–2.64	0.242
IMWG -FI			
Intermediate vs fit	0.00	0.00– .	0.999
Frail vs fit	0.00	0.00– .	0.999
R-MCI			
Intermediate vs fit	0.33	0.08–1.45	0.143
Frail vs fit	0.17	0.02–1.42	0.101

G8, Geriatric 8 scale; GAH, Geriatric Assessment in Hematology scale; Frail-VIG Index, VIG is the Spanish abbreviation for Comprehensive Geriatric Assessment; CFS, Rockwood's Clinical Frailty Scale; IMWG-FI, International Myeloma Working Group Frailty Index; R-MCI, Revised Myeloma Comorbidity Index.
